# A Survey of Smallholder Farms Regarding Demographics, Health Care, and Management Factors of Donkeys in Northeastern China

**DOI:** 10.3389/fvets.2021.626622

**Published:** 2021-04-14

**Authors:** Liang Deng, Shicheng Shi, Jing Li, Chi Tang, Yuwei Han, Peng Xie

**Affiliations:** ^1^Department of Animal Genetics, Breeding and Reproduction, College of Animal Science and Veterinary Medicine, Shenyang Agricultural University, Shenyang, China; ^2^Department of Clinical Veterinary Medicine, College of Veterinary Medicine, China Agricultural University, Beijing, China

**Keywords:** demographics, management, health care, survey, donkey, China

## Abstract

Essential information on the population dynamics and the health and welfare of Chinese donkeys is scarce. The objectives of this study were to describe the demographic characteristics, management and health care of a sample of donkeys under smallholder farm conditions of northeastern China. A cross-sectional survey of 731 randomly selected donkey owners on smallholder farms (1,658 donkeys) in 40 villages of northeastern China was conducted. Data on the composition and management of the donkeys and their routine health care were analyzed. The surveyed donkey population consisted of mostly (83.8%) jenny/filly donkeys with a mean age of 6.2 ± 5.0 years. Most (91.2%) of the farms kept 1–4 donkeys. The majority of donkeys were used for breeding and labor. Most (93.8%) of the farms did not have bedding, and their mean stable size was 17.7 ± 10.1 m^2^. All of the animals were turned out for at least part of the year. The mean size of the turnout areas on the farms was 17.8 m^2^. The condition of 12.5% of the donkeys was evaluated as “poor” with a body condition score of 1 on a scale of 5. More than one third (37.9%) of the donkeys had never been dewormed. Also, none of them were ever vaccinated or received dental care from a veterinarian. Their hoofs were trimmed once (45.9%) or twice (27.6%) a year. Forty percent of the donkeys were reported to suffer from at least one medical problem in the preceding year. The most common medical problems were colic, respiratory disorders and skin conditions. Owners seemed to underestimate some of the most prevalent diseases in donkeys, suggesting that their knowledge of the management of donkeys, including routine healthcare practices should be improved to ensure the health and welfare of donkeys in northeastern China.

## Introduction

The latest estimate of the global donkey population is 50.45 million, of which 2.68 million are raised in China (1, 2). Donkeys comprise a key animal species that has made valuable contributions to our society. They have a long history of serving various purposes in China, such as milk, meat and hide production, and labor and recreation (3).

Previous studies have revealed demographic characteristics of donkeys, such as the size and composition of the endangered Miranda donkeys in Portugal (4, 5), the donkey population in The Donkey Sanctuary of United Kingdom (UK) (6–8) and the working donkeys in developing countries (9, 10). These authors have described disproportionate or unstable distributions in age and gender among some groups of donkeys (9, 10) or the relatively long lifespans and good health of others (6, 7). Baseline demographic data on the general donkey population is essential to evaluate population dynamics (11), and the relevance and impact of outcomes of epidemiological control (12).

It is important to ensure appropriate management and routine care to maintain good health among donkeys. Different types of nutrition, stabling facilities, activity, environment, health care, and culture can influence the welfare, development of disease and average breeding life of horses and donkeys (7, 13–15). However, donkeys often suffer significant health problems owing to physiologic characteristics and stoical behavior which are often misinterpreted by owners (16). Donkeys often do not exhibit obvious clinical signs despite suffering from severe or even life-threatening conditions (17). The characteristics in behavior, physiology and health have been recognized by some donkey researchers, para-Governmental bodies and owners, who have developed management guidelines for disease prevention (18, 19) and applied improved management practices to control disease risks (15, 17).

More recently, the feasibility of establishing intensive donkey farming systems in China to supply the demand for hides has been investigated (11). More than 70% of donkeys in China are raised under extensive conditions on smallholder farms, which reflect the population dynamics of extensive donkey farming systems. Forty-five percent of all Chinese donkeys are raised, bred and used in northeastern China, an important donkey-breeding region, and most of them remain under smallholder farm conditions (2). Our recent study investigated the foaling-related parameters and dental disorders of jennies in this region (20, 21).

To the best of our knowledge, little information is known about the demographic characteristics, management and health care of donkeys in northeastern China. Hence, this cross-sectional study aimed to ascertain the demographic characteristics, management and health care of the extensively managed donkey population in northeastern China.

## Materials and Methods

### Study Area

We conducted this study in Western Liaoning Province and the Eastern Inner Mongolia Autonomous Region, two adjacent zones with numerous hills in northeastern China, at an altitude ranging from 300 to 1,200 m above sea level, between coordinates 118°50'–122°26' E and 40°17'–43°01' N. This part of China has a semi-arid climate with an average monthly maximum temperature 24.1°C in July and minimum temperature −10.1°C in January. Meanwhile, the average monthly maximum precipitation is 129.8 mm in July and minimum precipitation is 1.8 mm in January. The mean relative humidity is 51%. The study area is 290 km from north to south and 300 km from east to west: 37,500 km^2^ in total. The farmers practiced a mixed crop–livestock production systems, and used donkeys for breeding and light farm work. The donkey herds in the study area consisted of ~0.8 million indigenous Liaoxi donkeys described in our previous study (20), which account for two-thirds of donkeys in northeastern China. Breeding was practiced in a relatively extensive system. Thirty to fifty jennies were bred by in-hand breeding with natural service using a shared jack, following oestrus, usually observed and detected by the jenny owners.

### Study Methodology and Data Collection

A cross-sectional survey of 731 smallholder farms was conducted in 40 villages between March and July 2017 with face-to-face interviews of the donkey owners of each farm. The farms were selected using a stratified sampling method according to their geographical distribution (north, east and south areas). Among each area, the farms were randomly selected based on the density statistics of donkeys by local government. These farms were visited door to door, and owners were asked if they would like to take part in the survey, resulting in a convenience-based sample of owners who were willing to participate. The survey, which took ~20 min to complete, was combined with a pre-tested, semi-structured questionnaire, which was developed using a modification of the tailored design method (22). The questionnaire contained sections on the demographics of the owner (age, gender, educational level, source of income and the number of years spent raising donkeys), the donkey (age, gender, herd size and use), general management practices (stabling, turnout and feeding) and health care measures and interventions (body condition scores, medical problems during the preceding year, deworming, vaccination, dental care and hoof trimming) (Supplementary Table 1). A trained enumerator measured the size of the stables and turnout areas and rated the body condition of the donkeys on a 5-point scale (1 = poor, 2 = moderate, 3 = ideal, 4 = fat, and 5 = obese), in accordance with the guidelines of The Donkey Sanctuary (23). Photographs of the donkeys were collected to confirm the identity of each one. The total sample size was ~0.2% of the total number of households with donkeys based on the population census of 2016.

The data collection protocol was implemented with the approval of the Shenyang Agricultural University Animal Care and Use Committee (Permit no.: 201702018).

### Statistical Analysis

The baseline survey data were entered into Microsoft Office Excel 2007 (Microsoft Corp., Redmond, WA), then exported to SPSS version 22.0 (IBM Corp., Armonk, NY) after checking it for possible mistakes, typing errors and outliers. Descriptive statistics, including frequencies, means, medians, ranges, percentages and standard deviations (SDs) of the variables were calculated. The results are expressed as mean or mean ± SD and 95% confidence intervals (CI). Normality was assessed using the Shapiro–Wilk test. Comparisons between groups were made using Pearson's chi-squared test for categorical variables. Statistical significance was set at *P* <0.05.

## Results

### Demographic Characteristics of the Respondents

**Figure 1:**
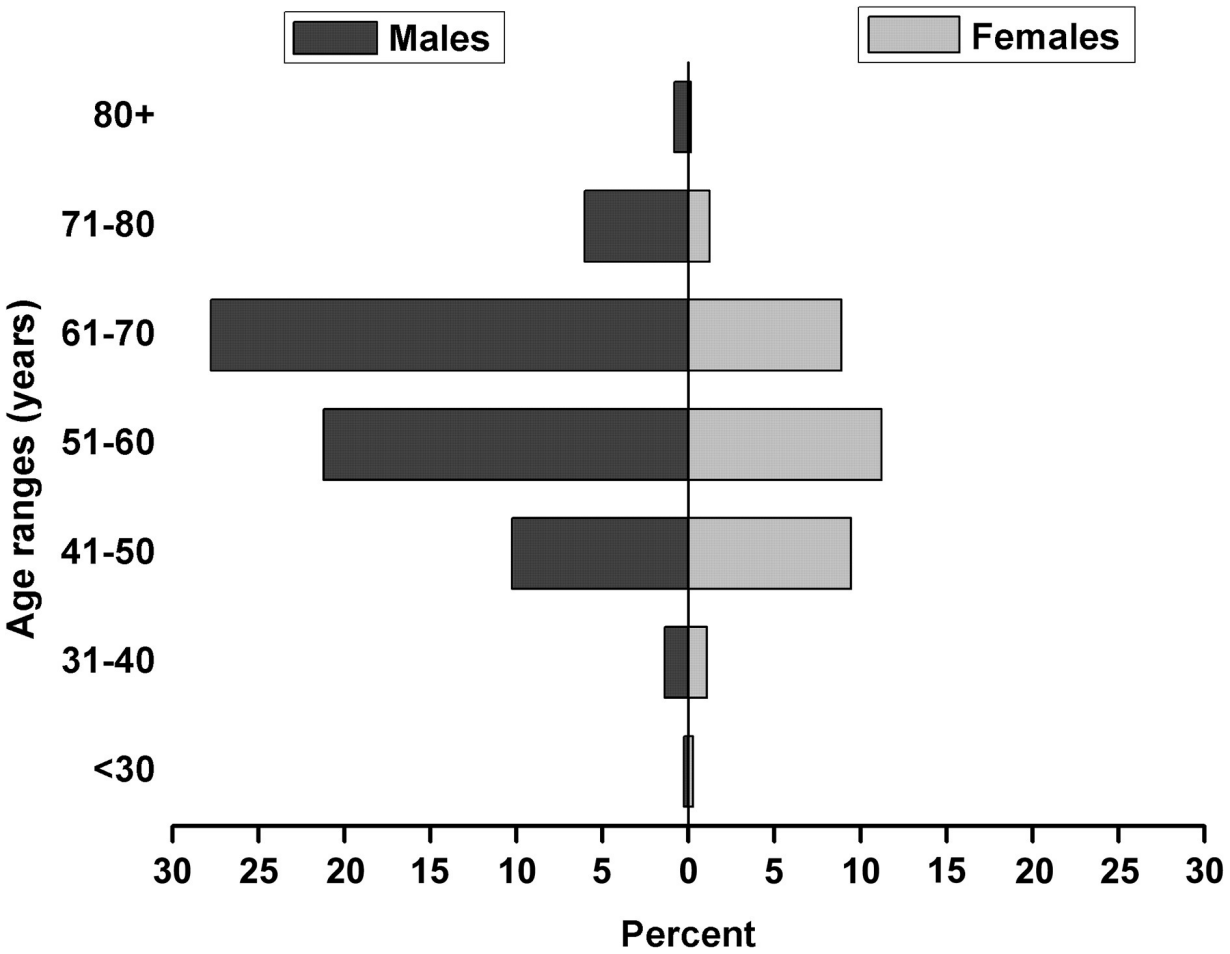
Age and gender distributions of the donkey owners in Western Liaoning and Eastern Inner Mongolia of China (*n* = 731).

A total of 731 donkey owners completed the survey. The age and gender distributions of the respondents are shown in Figure 1. Two-thirds of the respondents (67.7%, 95% CI: 64.3–71.1) were males and most of them (88.8%, 95% CI: 86.5–91.1) were 41–70 years old. The mean age of the respondents was 58.3 ± 9.5 years. The majority of respondents (96.6%) had ≤ 9 years of education, and nearly a half (48.6%) relied on agriculture as their all source of income. Most of them (81.5%) had raised donkeys for longer than 25 years (Table 1).

**Table 1:** Respondents (Donkey owners)' number of years of education, source of income, and years raising donkeys in Western Liaoning and Eastern Inner Mongolia of China (*n* = 731).

**Characteristic**	**Percentage (number) of respondents**	**95% CI**
**Education (years)**		
0	13.7 (100)	11.2–16.2
<3	10.0 (73)	5.7–14.3
4–6	37.9 (277)	34.4–41.4
7–9	35.0 (256)	31.5–38.5
10–12	2.9 (21)	1.7–4.1
>12	0.5 (4)	0–1.0
**Source of income**		
Agricultural activity	48.6 (355)	45.0–52.2
Agricultural activity + Employment	51.4 (376)	47.8–55.0
**Years raising donkeys**		
<5	2.9 (21)	1.7–4.1
6–10	2.5 (18)	1.4–3.6
11–15	3.7 (27)	2.3–5.1
16–20	3.4 (25)	2.1–4.7
21–25	6.0 (44)	4.3–7.7
>25	81.5 (596)	78.7–84.3

*CI, confidence interval*.

### Age and Gender Distributions of the Donkeys

**Figure 2:**
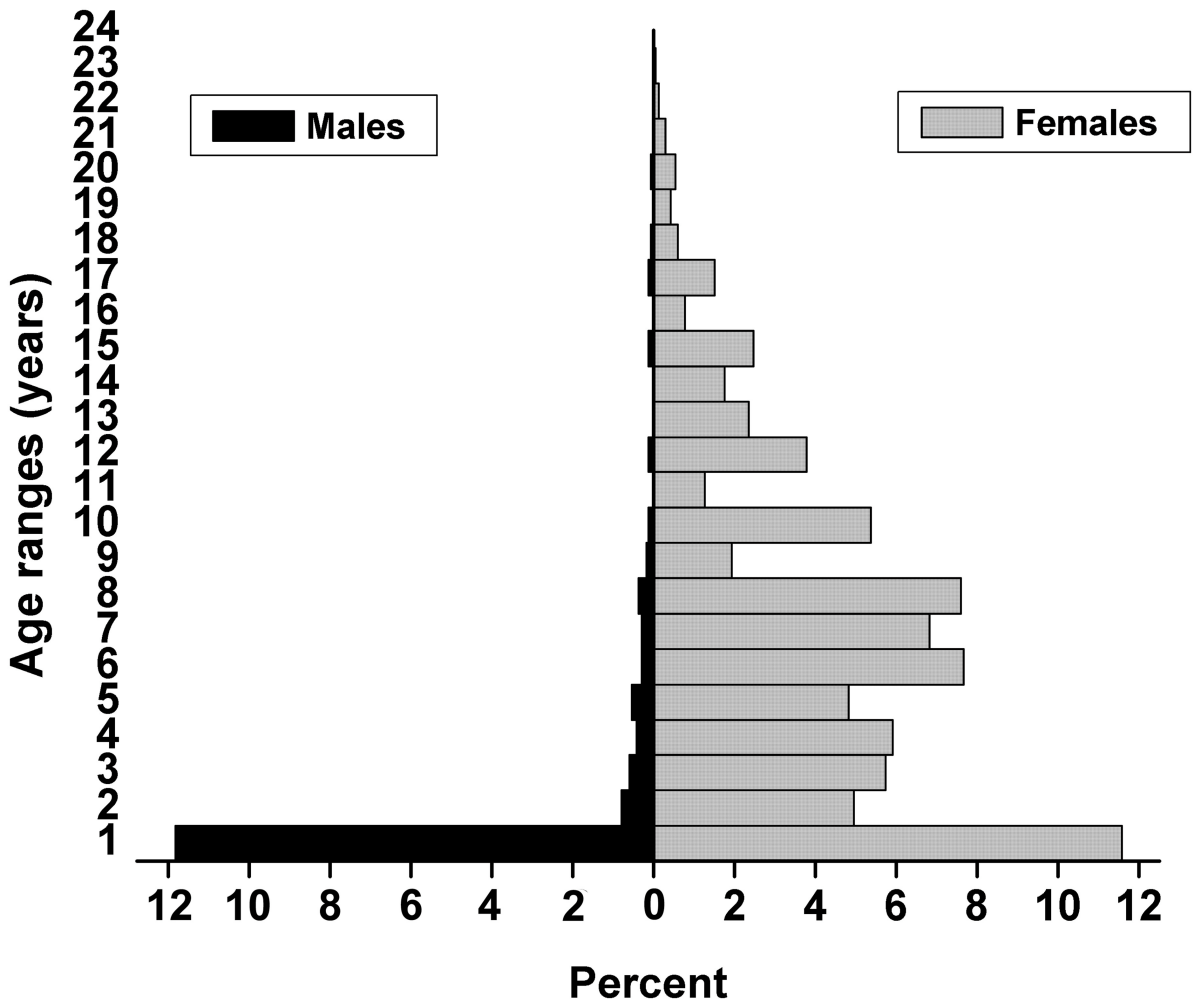
Overall age distribution of the donkeys by gender in Western Liaoning and Eastern Inner Mongolia of China (*n* = 1,658).

A total of 1,658 donkeys were included in the study. The herds mostly consisted of jenny/filly donkeys (83.8%, 95% CI: 82.0–85.6), jack/colt donkeys (15.9%, 95% CI: 14.1–17.7) and gelding donkeys (0.2%). The mean and median ages of the sample were 6.2 ± 5.0 years and 5 years (range: 0–23 years), respectively, and the largest and second largest number of age groups were ≤ 1 year (23.4%, 95% CI: 21.4–25.4) and 6–8 years (23.0%, 95% CI: 21.0–25.0), respectively. The number of females and males decreased dramatically after 1 years old. Among the 264 jack/colt donkeys, only 40 donkeys were over 3 years old and used for breeding, which account for a small percentage (2.4%) in this surveyed population (Figure 2).

### Herd Size

The herd size of the donkeys across the 694 smallholder farms is presented in Table 2. The average farm size was 1.7 ± 1.3 ha and most (91.2%) of the farms kept 1–4 donkeys. The mean herd size and mean number of breeding jennies per farm were 2.5 ± 1.5 and 1.7 ± 0.9, respectively. The herd to farm ratio and breeding jennies to farm ratio were 2.2:1 and 1.6:1, respectively.

**Table 2:** Herd size of donkeys across 694^*^ smallholder farms in Western Liaoning and Eastern Inner Mongolia of China.

**Herd and farm size**	**Mean**	**Median**	**SD**	**Range**
Farm size (ha)	1.7	1.3	1.3	0–12
Herd size	2.5	2	1.5	1–14
Breeding jennies size	1.7	1	0.9	0–10
Herd size/farm size (per ha)	2.2	1.5	2.1	0–22.5
Breeding jennies size/farm size (per ha)	1.6	1.2	1.6	0–22.5

*SD, standard deviation; ha. hectare*.

* *Thirty-seven respondents did not provide the information*.

### Uses of Donkeys

The majority of donkeys were used for breeding (79.7%), and a large proportion was used for work, such as driving (61.6%), packing (3.4%) and agricultural operations during drafts (69.1%). Only 11.4% of the donkeys were used for meat and hide production (Table 3).

**Table 3:** Uses of donkeys across smallholder farms in Western Liaoning and Eastern Inner Mongolia of China (*n* = 1,658).

**Uses**	Percentage (number) of donkeys*	**95% CI**
Breeding	79.7 (1322)	77.8–81.6
Draft	69.1 (1146)	66.9–71.3
Driving	61.6 (1022)	59.3–63.9
Production (meat and hide)	11.4 (189)	9.9–12.9
Packing	3.4 (56)	2.5–4.3
Not reported	0.8 (14)	0.4–1.2

*CI, confidence interval*.

* *More than one answer was allowed*.

### Stable Management

Overall, 99.5% of the donkeys were routinely stabled at some time throughout the year. The types of stable structures and bedding varied (Table 4). Most of the stables were remodeled houses in which owners lived previously (44.7%) and simple brick stables (40.8%). Others were made of wood with an iron roof (14.0%).

**Table 4:** Characteristics of the stables across smallholder farms in Western Liaoning and Eastern Inner Mongolia of China (*n* = 731).

**Stables**	**Percentage (number) of farms**	**95% CI**
**Type of structure**		
Remodeled from old houses	44.7 (327)	41.1–48.3
Simple brick stable	40.8 (298)	37.2–44.4
Wood stable with an iron roof	14.0 (102)	11.5–16.5
No stable	0.5 (4)	0–1.0
**Stable size (m**^**2**^**)**		
0	0.5 (4)	0–1.0
1–25	79.3 (580)	76.4–82.2
25–50	17.0 (124)	14.3–19.7
51–75	0.5 (4)	0–1.0
>75	0.3 (2)	−0.1 to 0.7
Not reported	2.3 (17)	1.2–3.4
**Type of bedding**		
No bedding	93.8 (686)	92.1–95.5
Straw	6.2 (45)	4.5–7.9

*CI, confidence interval*.

The mean and median stable size per farm was 17.7 ± 10.1 m^2^ and 14.8 m^2^ (ranges 0–103 m^2^), respectively. Among these farms, there were four (0.5%) kept their donkeys in the yard without any stable or shelter throughout the year. Most of the farms stabled the donkeys in areas measuring 1–25 m^2^ (79.3%), followed by 25–50 m^2^ (17.0%). Most of the farms did not have bedding (93.8%), and only a small number of donkeys had straw bedding (6.2%).

### Turnout Management

The types and sizes of the turnout areas are summarized in Table 5. In the majority of turnout areas, a donkey was tied to a pole outside (39.0%), followed by a turnout to a paddock without grass (24.2%) and a walking area in a yard (8.3%). A small proportion of farms (5.3%) had a pasture with an access for donkeys.

**Table 5:** Types and sizes of turnout areas across smallholder farms in Western Liaoning and Eastern Inner Mongolia of China (*n* = 731).

**Turnout areas**	**Percentage (number) of farms**	**95% CI**
**Type of turnout area**		
Pole tied	39.0 (285)	35.5–42.5
Paddock without grass	24.2 (177)	21.1–27.3
Yard	8.3 (61)	6.3–10.3
Pasture	5.3 (39)	3.7–6.9
Not reported	23.1 (169)	20.0–26.2
**Turnout area size (m**^**2**^**)**		
<25	53.9 (394)	50.3–57.5
25–50	16.4 (120)	13.7–19.1
51–75	4.8 (35)	3.3–6.3
>75	1.8 (13)	0.8–2.8
Not reported	23.1 (169)	20.0–26.2

*CI, confidence interval*.

The mean and median size of the turnout areas were 17.8 and 13.4 m^2^ (ranges 1.7–145 m^2^), respectively. Most of the donkeys were kept in small turnout areas measuring ≤ 50 m^2^ (70.3%); only 1.8% of the farms kept donkeys in turnout areas larger than 75 m^2^.

The durations of the turnouts varied by season (Table 6). The majority of donkeys (73.7–95.0%) were turned out during the day and stabled at night, throughout the year. A larger proportion was turned out 24 h/day without access to any stable during the summer (26.3%) and spring (22.6%), compared to the other seasons (0.8–16.6%, *P* <0.01). A small proportion of donkeys were stabled 24 h/day during the winter (4.3%) and autumn (0.8%). Overall, the donkeys' median turnout duration was 10 h/day, although there was seasonal variation: a median of 12 h/day in the summer, 11 h/day in the spring, 10 h/day in the autumn and 8 h/day in the winter.

**Table 6:** Stable and turnout management of donkeys across smallholder farms in Western Liaoning and Eastern Inner Mongolia of China throughout the year (*n* = 1,585).

	**Percentage (number) of donkeys**			
	**Time of year**			
**Turnout**	**Sept–Nov**	**Dec–Feb**	**Mar–May**	**Jun–Aug**
Night stabled, day turned	81.4 (1290)^b^	95.0 (1505)^a^	77.4 (1226)^bc^	73.7 (1168)^cd^
out				
Turned out 24 h/day	16.6 (263)^b^	0.8 (12)^c^	22.6 (359)^a^	26.3 (417)^a^
Stabled 24 h/day	2.0 (32)	4.3 (68)	0	0
Median time spent outside	10	8	11	12
(h/day)				

*a−d Lowercase superscript letters indicate a significant difference (P < 0.01)*.

### Dietary Management

Donkeys were mainly fed crop residue, such as millet straw (85.4%) and maize straw (83.1%), whereas 33.0% were fed hay (Table 7). More than half of the donkeys were offered forage twice daily (52.6%) and 32.2% were fed forage daily *ad libitum*.

**Table 7:** Dietary management provided for donkeys across smallholder farms in Western Liaoning and Eastern Inner Mongolia of China (*n* = 1,658).

**Feed type and feeding frequency**	**Percentage (number) of donkeys**	**95% CI**
Type of forage feed*		
Millet straw	85.4 (1416)	83.7–87.1
Maize straw	83.1 (1378)	81.3–84.9
Hay	33.0 (547)	30.7–35.3
Alfalfa	0.7 (12)	0.3–1.1
Other	0.5 (8)	0.2–0.8
Not reported	0.4 (7)	0.1–0.7
**Frequency of forage feeding daily**		
1 time/d	4.1 (68)	3.2–5.1
2 times/d	52.6 (872)	50.2–55.0
3 times/d	10.7 (177)	9.2–12.2
Ad libitum	32.2 (534)	30.0–34.5
Not reported	0.4 (7)	0.1–0.7
Type of concentrate*		
Maize	89.2 (1479)	87.7–90.7
Soybean meal	16.9 (280)	15.1–18.7
Sunflower seed meal	14.8 (245)	13.1–16.5
Wheat bran	6.9 (114)	5.7–8.1
Mineral or vitamin supplements	29.0 (481)	26.8–31.2
Commercial products	0.7 (12)	0.3–1.1
Other	11.3 (187)	9.8–12.8
None	0.2 (3)	0–0.4
Not reported	0.4 (7)	0.1–0.7
**Frequency of concentrate feeding daily**		
1 time/d	24.7 (410)	22.6–26.8
2 times/d	51.1 (848)	48.7–53.5
3 times/d	23.5 (390)	21.5–25.5
None	0.2 (3)	0–0.4
Not reported	0.4 (7)	0.1–0.7
**Water availability**		
Automatic drinker	5.5 (92)	4.4–6.6
Bucket	94.5 (1566)	93.4–95.6
**Cleanliness of water**		
Clean	72.3 (1198)	70.2–74.5
Dirty	27.7 (460)	25.6–29.9

*CI, confidence interval*.

* *More than one answer was allowed*.

Most of the donkeys' diets were supplemented with small amounts of homemade concentrates, including maize (89.2%), soybean meal (16.9%), sunflower seed meal (14.8%) and wheat bran (6.9%). Only 29.0% of the donkeys were given vitamin and mineral supplements, and 0.7% were fed commercial products. Three donkeys (0.2%) were fed without concentrates. Over half (51.1%) of the donkeys' diets were supplemented with concentrates twice daily, 24.7% were supplemented once daily and 23.5% were supplemented three times daily.

All the donkeys were provided with water daily, 5.5% automatic drinkers and 94.5% bucket. There were 72.3% donkeys could drink clean water, although remains with dirty water.

### Body Condition Score

The BCS was ideal in the largest proportion of the donkeys (43.2%, 95% CI: 40.8–45.6), moderate in 27.7% of them (95% CI: 25.5–29.9), fat in 15.8% (95% CI: 14.0–17.6%), poor in 12.5% (95% CI: 10.9–14.1%) and obese in 0.8% (95% CI: 0.4–1.2%) of the donkeys.

### Health Care

The provision of routine health care to the donkeys, such as deworming, vaccination, dental care and hoof trimming, were examined (Table 8). More than one third (37.9%) of the donkeys were not dewormed yearly. Nearly half (44.5%) were dewormed once per year and 12.4% were dewormed twice per year. None of the donkeys in this study had ever been vaccinated or received dental care from a veterinarian or a dental technician. A farrier performed hoof trimming of the animals without being shod. A total of 761 (45.9%) and 458 (27.6%) donkeys had their hoofs trimmed once and twice a year, respectively. Only 20 donkeys (1.2%) had their hoofs trimmed three times a year; however, 245 donkeys (14.8%) had never had their hoofs trimmed.

**Table 8:** Health care of donkeys across smallholder farms in Western Liaoning and Eastern Inner Mongolia of China (*n* = 1,658).

**Type of healthcare**	**Percentage (number) of donkeys**	**95% CI**
**Frequency of deworming**		
Never	37.9 (628)	35.6–40.2
Once per year	44.5 (738)	42.1–46.9
Twice per year	12.4 (205)	10.8–14.0
More often than every 4 months	0.4 (7)	0.1–0.7
Dewormed, depending on fecal examination	2.7 (45)	1.9–3.5
Not reported	2.1 (35)	1.4–2.8
**Vaccinations**		
Not vaccinated	100 (1658)	
**Dental care**		
Never by a veterinarian or dental technician	100 (1658)	
**Frequency of hoof trimming**		
Never	14.8 (245)	13.1–16.5
Once per year	45.9 (761)	43.5–48.3
Twice per year	27.6 (458)	25.4–29.8
Three times per year	1.2 (20)	0.7–1.7
Not reported	10.5 (174)	9.0–12.0

*CI, confidence interval*.

### Common Medical Problems

The most common medical problems of the donkeys during the preceding year are presented in Table 9. Most (60.5%) of the donkeys did not suffer from any medical problems in the preceding year. Among those that did have medical problems, 13.5% had colic, 10.9% had respiratory disorders, 9.2% had skin disorders, 3.4% had lameness and 2.7% had oral/dental disorders, which were considered common issues.

**Table 9:** Common medical problems of donkeys across smallholder farms in Western Liaoning and Eastern Inner Mongolia of China (*n* = 1,658).

**Medical problems**	Percentage (number) of donkeys*	**95% CI**
None	60.5 (1003)	58.1–62.9
Colic	13.5 (224)	11.9–15.1
Respiratory disorders	10.9 (181)	9.4–12.4
Skin disorders	9.2 (153)	7.8–10.6
Lameness	3.4 (56)	2.5–4.3
Oral/dental disorders	2.7 (45)	1.9–3.5
Other^+^	4.4 (73)	3.4–5.4
Not reported	7.4 (123)	6.1–8.7

*CI, confidence interval*.

* *More than one answer was allowed*.

+ *Other common medical problems including tetanus, intoxication, septic foals, colitis, body mass, and abortion from unknown origins*.

## Discussion

The present study was designed to provide a description of the demographic characteristics, management and health care of a large cohort of donkeys under smallholder farm conditions in northeastern China. To the best of our knowledge, this is the first comprehensive survey of donkey owners using face-to-face interviews and questionnaires to explore the status of a Chinese donkey population. Our analysis and evaluation of the data collected should be valuable in providing baseline reference information for future studies and improving the health and welfare of donkeys in China.

The farm sample for this cross-sectional survey was selected using a stratified sampling method by visiting the region and owners participated on a voluntary basis. However, the willingness of the owners to participate could be a bias toward the more caring and candid donkey owners. In our study, over 90% owners we visited were glad to participate in the survey; therefore, the sample could be representative of the population. The face-to-face method of obtaining data enhanced the respondents' participation and minimized nonresponses and errors, compared with previous surveys using only questionnaires related to donkeys (7) and horses (14, 24).

In this study, the majority of owners who raised donkeys were males, similar to the farmers who used working equines in the campesino hill-slope communities of central Mexico (25). The mean age of the owners was 58.3 ± 9.5 years, which was 7 years younger than that of the owners of the Miranda donkey herds in Portugal (5). The advanced age of the owners suggest a further decrease in the number of donkey herds in the near future (5). In terms of educational level, only a small proportion of owners had completed junior high school. Thus, lack of formal education could have affected the management and health care of their donkeys.

The mean age of the donkeys herein (6.2 ± 5.0 years) was similar to that of the market donkeys reported in central Ethiopia (26) and working donkeys on family farms in Albania (27). Nevertheless, they were significantly younger than the donkeys with a mean age >20 years in foster care (7), those raised in The Donkey Sanctuary in the UK (6) and those working in Zimbabwe (9). The mean age revealed a significantly younger and premature culling of donkeys in China, owing to the potential for nutritional stressors, improper health care and production use. This study's sample had a significantly higher proportion of female than male donkeys. Similar results were found in Nigeria (10) and Portugal (4), although an equal distribution of males and females was reported in Mexico (28). Furthermore, female donkeys younger than 1 year accounted for ~12% of the current sample. To maintain the herd size, it would be reasonable for owners to have at least 25% pubescent females for their herds (5). In contrast to the results found in other countries, in this study a very small proportion (2.4%) of males was maintained in the herd as breeders and almost all males were sold during early weaning to the intensive feedlot for meat and hide purpose. Therefore, the low ratio of siring males could have resulted in the unequal contribution to the genetic pool of the population (5).

Some owners had not fully embraced the importance of the proper housing of donkeys. The use of a wooden shelter with an iron roof and no stable requires a critical evaluation because these housing features could be associated with stress caused by cold temperatures, especially in the early hours of the day in the winter (29, 30). The amount (93.8%) of donkeys stabled without bedding differed significantly from donkeys in The Donkey Sanctuary, where only 5.0% were reported to have no bedding (7). Fifty-five percent of facilities provided clean bedding to donkeys in Italy and UK (30). Lack of bedding could be detrimental to donkeys, which may suffer from cold-related stress (30). Straw is the most frequently (75%) used bedding type for donkeys (7). Horses on straw bedding have been found to spend three times longer in a lateral recumbent position overnight than they did on shavings, suggesting a beneficial health effect of straw (31).

Most of the donkeys in this study were stabled at night and turned out during the day, throughout the year. The mean stable size (17.7 ± 10.1 m^2^) probably was inadequate considering the mean herd size per farm was 2.5 donkeys. One donkey should be stabled in a box ~3.3 ×3.3 m (32) or provided with the suitable box according to the body size, to provide a healthy and safe environment. The mean size of the turnout areas in this study, were consistent with the recommended dimension of 7 m^2^ per donkey for milk production (19). The duration of the daytime turnouts varied between seasons, consistent with previous studies of donkeys (7) and horses (14, 33, 34), indicating that time spent outside is likely to affect equine health and welfare (7) by providing opportunities to exercise (35), avoiding the risk of stereotypies (36) and reducing exposure to respiratory allergens within the stable environment (33). However, there was no regular turnout for grazing in this study; herein, pastures were scarce due to the semi-arid climate.

Consistent with the recommendations for feeding donkeys by The Donkey Sanctuary (37), all of them were provided with forage, the main source of high-fiber roughage, such as millet straw and maize straw, reflecting the types of products readily available in northeastern China. One third of the donkeys were fed with hay, which is the most frequently used forage feed for donkeys in The Donkey Sanctuary (7) and for horses in various countries (14, 33, 38, 39). In addition to the forage, most of the donkeys were fed some concentrates/supplementary feed; working donkeys may require supplementary feeds due to the increased energy requirement. Concentrates are required and should be low in cereal grain content in order to avoid related health problems (40). Nevertheless, owners' basic knowledge of donkey nutrition was inadequate (20), resulting in a diet with nutrient deficiencies and/or dietary excesses. Most of the donkeys in our study were taken to clean water by bucket during the day, while 15 and 20% donkeys were provided with dirty and very dirty water in 20 donkey facilities in Europe, respectively (30). The general rule is that donkeys should always be provided with free access to clean water throughout the day (41).

Body condition scoring is a useful tool for fine-tuning diets to a donkey's individual requirements (37). In the present study, most of the donkeys maintained an ideal BCS, as they were mainly involved in breeding and light farm work. Additionally, the condition of 12.5% of the donkeys was rated as poor and 27.7% as moderate, which was more than 0.36% (poor) and 6.85% (moderate) of donkeys in 20 donkey facilities in Europe, respectively (30). Working donkeys with a low BCS may have comorbid dental disease, liver disease, gait abnormalities and other health issues (42, 43). Thus, it is important to promote energy intake, dental care, parasites control and general health care throughout the day to improve a donkey's body condition. In contrast to the conditions in which 33.8% of the donkeys were overweight or obese, as described in The Donkey Sanctuary (7), only a few of the donkeys in our study could be considered overweight.

The frequency of preventive healthcare interventions, such as deworming, vaccination and dental and hoof care, was low in the current study, which was similar to the frequency reported for working donkeys in most developing countries (17, 44).

In this study, more than one third of the donkeys were never dewormed. Without anthelmintics, the average life of a donkey varies from 9 years in Ethiopia to 15 years in Mexico (45). Over half of the donkeys in this study were dewormed once or twice per year. A mean annual deworming frequency consisting of 2 treatments has beneficial effects on the performance (health, longevity, and ability to work) of donkeys. In Ethiopia, the most suitable time for deworming is at the end of the dry and wet seasons (45).

None of the donkeys in this study was reported to be vaccinated against any disease. To date, no domestic commercial vaccines are available for horses or donkeys in mainland China, except some commercial vaccines imported and used for sport horses. Infectious equine disease in donkeys has been found within China. For example, an equine influenza outbreak on a 300-head donkey farm in Shandong Province resulted in a 25% mortality rate (46), which was also reported to be a common occurrence. In addition, the increase in abortions due to *Salmonella abortus equi* was reported on a farm with over 1,000 jennies (47). Thus, donkeys should be included in vaccination programs promoted by the government to enhance herd immunity and reduce individual risk.

The 2.7% prevalence of dental disorders reported by the owners was based on their inadequate knowledge. However, all the respondents confirmed that none of the animals had received routine dental care by a veterinarian or dental technician. Large populations of adult donkeys with no dental care and moderate-to-severe dental disease are common in China (21). Major dental disorders in geriatric donkeys (48) could be prevented or managed more effectively if they received routine dental care from an early age. Poor dentition and infrequent dental treatment were associated with colic in donkeys (6, 49). The prevalence of dental disease in China may be underestimated, similar to The Donkey Sanctuary in the UK (7), given the few clinical signs exhibited by donkeys, the limited number of equine veterinarians and dental technicians and the lack of donkey owners' knowledge concerning specific dental problems. More recently, we revealed that the most common dental disorders of jennies were sharp enamel points, incisor diastemata and focal overgrowths in Liaoning Province of China (21); therefore, proper dental care should be provided to improve the welfare of donkeys in China.

Hoof disease and hoof-related lameness is common in donkey populations worldwide. Donkeys are almost four times more likely to have sole abnormalities than are horses or mules (42). Lameness was reported in 3.4% of the donkeys during this study, which was significantly lower than the 27.2% prevalence of lame donkeys at The Donkey Sanctuary in the UK (50), 18.7% of the dairy donkeys' hoofs neglect in Italy (51), and 100% of the lame working draft donkeys in Pakistan (52). The prevalence of hoof disease in this study could be underestimated, owing to the donkey owners' lack of knowledge concerning the diagnosis of hoof disease. All the donkeys in this study were trimmed three or fewer times per year, which is significantly lower than the requirement that donkeys' hoofs be trimmed every 6–10 weeks (50). In this study, the percentage of donkeys reported to have never had hoof care was less than the 40% of donkeys reported in The Donkey Sanctuary in the UK (7).

A study in the UK reported that 59% of the donkeys suffered from at least one medical problem (7). However, only 39.5% of the donkeys were reported to have health issues in our study. The most common problems were colic and respiratory and skin disorders, which were more variable compared with studies of donkeys (7) and horses (14, 38, 39) in different countries. These differences may be due to the under-reporting of health issues in the present survey, which relied on the respondent's recall, compared to the medical records used by the veterinarians (34, 38). The traditional system of Chinese University education for army and working equine veterinarians has disintegrated (3); therefore, the existing equine veterinary services in mainland China are insufficient and lack routine data entries on donkeys into the medical records, owing to the small, scattered population of equine veterinarians with little or no formal education or training. Furthermore, it is possible that donkeys often do not exhibit obvious clinical signs when they experience disease. Thus, the recognition of some health problems by owners may be incorrect or inadequate, particularly chronic pain (17).

Colic is considered the third most important disease of horses in northern Britain (14), although it is ranked much lower on the list of common medical problems of donkeys in The Donkey Sanctuary in the UK (7). Our study revealed that the incidence of colic was 13.5%, making it the most common medical problem of the donkeys. All the cases were reported by the donkey owners; they were not diagnosed by veterinarians. The disease that caused the colic was unclear, considering the inadequate knowledge about its diagnosis. The incidence was higher than the incidence (3.9–5.9%) found in the donkeys in other studies (6, 49). It is more likely that the donkeys in this study stabled for half a day and were fed inappropriate concentrate feed or reduced water intake. Therefore, the colic that commonly occurred was similar to that reported in stabled horses (53).

## Conclusion

The results of this study provide valuable baseline information on the demographics, management and health care of the donkey population in northeastern China. A significantly younger and premature culling of the donkeys and a low ratio of siring males probably had a dramatic impact on the donkey population of China. Inadequate knowledge and ineffective management affected the health and welfare of the donkeys. The general information obtained in this study permits exploration of the interaction between managerial factors and health to improve the welfare of donkeys and build an appropriate farming system of donkeys in the future.

## Data Availability Statement

The original contributions presented in the study are included in the article/Supplementary Materials, further inquiries can be directed to the corresponding author/s.

## Ethics Statement

The animal study was reviewed and approved by Shenyang Agricultural University Animal Care and Use Committee.

## Author Contributions

LD performed the survey. SS, CT, and JL helped with data collection. YH and PX participated in data analysis. LD and JL edited the manuscript. All authors read and approved the final manuscript.

## Conflict of Interest

The authors declare that the research was conducted in the absence of any commercial or financial relationships that could be construed as a potential conflict of interest.
