# Supplementary material for: A Survey of Smallholder Farms Regarding Demographics, Health Care, and Management Factors of Donkeys in Northeastern China
Source: Front Vet Sci. 2021 Apr 14;8:626622. doi: 10.3389/fvets.2021.626622 (PMC8079733; doi:10.3389/fvets.2021.626622)
Supplement: Supplementary file 1 [file Table_1.docx]

Supplementary Material

# Supplementary Tables

**Supplementary Table 1.** Survey used for data collection.

**Questionnaire**

Name of the location

Date

Part 1. Herd information:

1. How many donkeys in your farm?

2. Sex/gender per donkey?

3. Age per donkey?

4. How many donkeys in your farm?

5. How many breeding jennies in your farm?

6. Which size of your farm?

7. Select the primary role of the donkey

□Breeding □Draught □Driving □Packing □Production (meat and hide) □Other (please specify)

Part 2. Management information:

1. Select the type of housing structure

□No housing □Wooden shelter combined by iron roof □Simple brick stable □Remodeled from old houses □Other (please specify)

2. Select the type of bedding

□No bedding □Straw □Wood shaving □Rubber matting □Other (please specify)

3. Dimensions of the stable?

4. Select the type of turnout area

□Yard □Pole tied □Paddock without grass □Pasture □Other (please specify)

5. Dimensions of the paddock?

6. How long do your donkeys turned out daily in each season?

7. Select the type of forage feed

□Millet straw □Maize straw □Hay □Alfalfa □Other (please specify)

8. How often do your donkeys have access to forage daily?

□1 time/d □2 times/d □3 times/d □Ad libitum

9. Select the type of concentrate feed

□None □Maize □Soybean meal □Sunflowerseed meal □Wheat bran □Mineral or vitamin supplements □Commercial products □Other (please specify)

10. How often do your donkeys have access to concentrate daily?

□None □1 time/d □2 times/d □3 times/d

Part 3. Health care information:

1. How often do your donkeys dewormed per year?

□Never □Once □Twice □Thrice □4 times □6 times □Deworm depending on fecal examination

2. How often do your donkeys have vaccinations per year?

□Never □Once □Twice □More than thrice

3. How often do your donkeys have dental care per year?

□Never □Once □Twice □More than thrice

4. How often do your donkeys have hoof trimmed per year?

□Never □Once □Twice □Thrice □More than 4 times

5. Which medical problems of your donkeys suffered from in the preceding year?

□None □Oral/Dental disorders □Respiratory disorders □Colic □Skin disorders □Lameness □Other (please specify)

Part 4. Respondent information:

1. Your gender?

2. Your age?

3. What is your education level?

4. What is your current job role?

5. What is your source of income?

6. How many years have you worked with donkeys?
